# Supplementary material for: Cardiovascular risk in newly diagnosed type 2 diabetes patients in India
Source: PLoS One. 2022 Mar 31;17(3):e0263619. doi: 10.1371/journal.pone.0263619 (PMC8970505; doi:10.1371/journal.pone.0263619)
Supplement: S1 Fig — The stripes in the box plots represent median score, the dots represent average values as presented. (PDF) [file pone.0263619.s001.pdf]

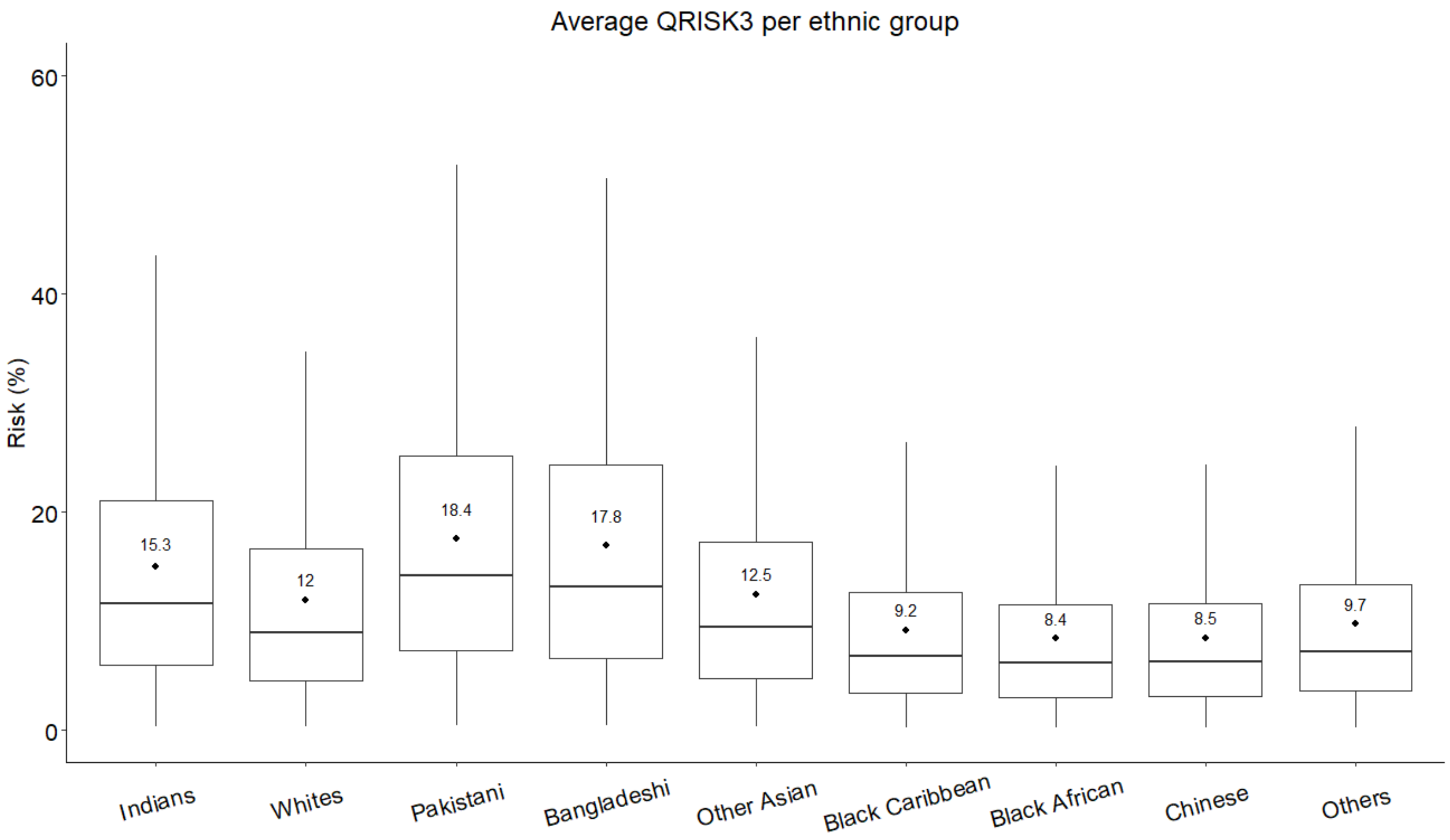

Figure S1

To determine the CV risk of the Indian ethnicity specifically as compared to other ethnicities, the data of the current 5,080 patients were used, only ethnicity was changed, and this was repeated for all ethnicities available in the QRISK3 model. Thereafter, the average CV risk was calculated for each ethnicity.
