# Supplementary material for: Cardiovascular risk in newly diagnosed type 2 diabetes patients in India
Source: PLoS One. 2022 Mar 31;17(3):e0263619. doi: 10.1371/journal.pone.0263619 (PMC8970505; doi:10.1371/journal.pone.0263619)
Supplement: S1 File — (DOCX) [file pone.0263619.s002.docx]

Names of participating doctors

| **Appendix** |  |  |  |  |  |
| --- | --- | --- | --- | --- | --- |
| **List of investigators:** | |  |  |  |  |
| Ashok Jain | Rajita | Shrikrishna V Acharya | Agasti Jawlekar | Utpal Roy Chowdhury | Mohanjeet Kaur |
| H P Sonania | Ramakrishana Reddy | Shripathi Rao | Ajay Bulle | Bidyut Kr Borah | Navtej Singh |
| Hemant Mahajan | S Krishna Mohan | Sm Suresh | Amol Ghumal | Bikash Bhattacharjee | R K Saproo |
| Kirnesh Pandey | Sanjay Paul | Sunil Kumar | Anil Kambale | Gautam Baral | Sandeep Chatwal |
| Manish Lashkare | Sainath Reddy | Sunil Kumar | Ashish Bhansali | Guru Prasad Bhattachrya | Seemant Garg |
| Rajendra Chowda | Syed Athar Hussain | T S Reddy | Ashish Kakria | Haobam Surjitkumar Singh | Shalu Gupta |
| Sanjay Shrivastav | Sysmala Aiyangar | Vedha Prakash | B D Wagh | Himanshu Nagar | Sunil Abrol |
| Sonal Dhaketa | Shehzad Ruman | Venkatesh C R | B R Malu | K K Barman | Surjit Singh |
| A K Jain | T Madhu Sudhan | Vijaykumar Shet | Badrinaryan Kshirsagar | Mahuya Sikdar | B S Bhatia |
| A R Pawar | Srirang Abkari | Y G Ashok Kumar | Bhushan Mapari | Mary Dcruz | Badrinath |
| Abhay Kumar | T Sathish | Abhay Gundgurthi | Devayani R Buche | Monoj Pandey | Gyaneshwar Maini |
| Abhinav Parikh | V Chandrashekar | Abhijit Bhograj | Dilip Dhope | Nadeem Akhter Khan | S S Bhatia |
| Ajay Singh | Aitha Akhila Reddy | Anantharaman | Dilip Thombre | Partha Pratim Sarma | Ab Hamid Zargar |
| Anil Shrivastav | Ashok Venkatanarasu | Chitra S | Dinesh Desale | Pradeep Dutta | Akshata Desai |
| Anish Siddiqui | Chaitanya Konda | D M Mahesh | Dipak Arun Khore | Ranjan Bhowmik | Bashir Ahmad Laway |
| Anupama Dubey | D Rajitha Reddy | K M Suryanarayana | Dipak Bahekar | Ripun Borpuzari | Emmy Grewal |
| Ashok Arora | G Kalyan Chakravarthy | Kishan Delampady | Dipak Deore | Sabyasachi Bandyopadhyay | Hayat Bhat |
| Ashok Tiwari | K D Modi | Praveen Ramachandra | Dipak Jaiswani | Sampat Jain | Hilal Mohiuddin Bhat |
| B P Bajpai | K Neelaveni | Santosh H S | Diwakar Gulajkar | Sumalya Sen | Javaid Rasool Bhat |
| Deepak Sharma | K Sandhya Rani | Shrinath P Shetty | G M Chandak | Sushanta Kumar Sen | K P Singh |
| Dinesh Garg | N Sudhakar Rao | Sudeep Putta Manohar | Ganesh Wadgaonkar | Ajitish Roy | Neeraj Garg |
| Girish Ramole | P Radha Rani | Vageesh Iyer | Gira R Soni | Amarta S Chowdhary | Nitin Gupta |
| Gopal Batni | Prasun Deb | Vijaya Sarathi H A | Jeet Sing | Amitabh Sur | Paramjit Singh Saini |
| H S Gajbhaiya | Ravi Kumar Muppidi | Rajiv Tungare | Jeetendra Gupta | Animesh Maiti | Rajesh Jain |
| J P Choudhary | Samantha Sathya Kumar | Yatin Gadgil | Jyoti Gajre | Biplab Mandal | Rakesh Goyal |
| Kalyan Sengupta | Shravan Ankathi | Amruta Prabhu | Kiran Kumavat | Chanchal Das | Ramanbir Singh |
| Mahendra Tilkar | Sreedevi Patnala | Avais Pathan | Kuldeep Raul | Indira Maisnam | Savita Jain |
| Mahesh Uppadhayoy | V Madhavi | Deepak Patil | M Kulkarni | Kaushik Biswas | Shariq Ahmed Masoodi |
| Manoj Indulkar | Vinay Kumar Dhanpal | Dilip Shah | Mahesh Khairnar | Pradip Chakraborty | Shweta Bansal |
| Manoj Mishra | Ch Bhaskar Rao | Fahad I Merchant | Manoj G Jahwar | Rachna Majumder | Siddharth Bindroo |
| Manoj Soni | P Kesava Rao | Girish Vaswani | Manish Bothara | Sayantan Ray | Monica Mahajan |
| Mukesh Bhargav | Y Uma Maheswararao | Kashyap Thakkar | Manish Choubey | Soumik Ghoswami | R Paul |
| Omesh Nandanwar | B R K Reddy | Kinnary Shah | Manish Pardeshi | Soumyabrata Roy Chaudhuri | Shailendra Gaur |
| Parvej Kamal | B Sudheer Kumar Reddy | Madhu Gupta | Manoj Barde | Subhodip Pramanik | V K Goyal |
| Prafull Dube | D Ramesh | Milind Katta | Manoj Nichat | Sunetra Mondal 00220764 | Sanjay Gogia |
| Rajeev Kapoor | G Rajani | Mitali Joshi | Mayur Punjabi | Tapas Chandra Das | Amardeep Sachdeva |
| Rajesh Agarwal | Meenaxisundari | Neha Sukthankar | Mayura Kale | A Sridhar Reddy | Ansuman Dalbehera |
| S S Gupta | P Satish Kumar Raju | P B Mody | Mrugraj Murtadak | M Vijay Kumar | Chetan Rijhwani |
| Sanjay Ambore | P Shanthi | Purvi Chawla | Mukund Ganeriwal | Shyam Prasad | I J Kalra |
| Subroto Biswas | Rakesh Kumar Vegesna | Ragini Maheshwari | N K Puranik | A Shashank | Lakhan Patel |
| Suri Virendra | Sahaja Aluru | Ravindra Bankapur | Nitesh S Chajed | Anil Kumar | M L Kalra |
| V Deewan | Shankar Reddy | Subhashree Patil | Nitin Raymule | B Maheshwar | Manas Chakravarty |
| V K Goyal | Sk Bhagawan | Tanuja Shah | Nitin Shinde | B Vinusha Reddy | Manish Kumar Sharma |
| Vinod Jain | A Venkatamuni | Anil Kumar M Singal | Parikshit S Baviskar | Bhaskar Rao | Manoj Ranka |
| Vinod Kothari | Arvind Sri Ram | Anurag Modi | Paritosh Jaisawal | C Hemanth | P K Sibbal |
| Vinod Porwal | Azmal Hussain | Ashish Deshmukh | Pitambar Digale | C R Ramreddy | Pankaj Sayyal |
| Yogendra Malhotra | B Nageswara Rao | Ashok Surana | Pragnesh Shah | Chaitanya Reddy | S P Kalra |
| A K Tiwari | C V Subramanyam | B D Kate | Pranesh Sanap | D Aravind Kumar | Sumedha Chhibber |
| Ashok Sethia | Ch Lakshmi Kiranmai | Heena Trehan | Pravin Nitnaware | Deepak | Sunil Wadhwa |
| B K Sethia | D V V Kesava Raju | Khadija Challawala | Prashant Somani | G Seshacharya | V K Malhotra |
| Bhupendra Ratra | Fazular Rahim | M K Dave | Purushottam Dadh | J Suresh | Anil Adya |
| M J Badwani | K Buchi Babu | Manjusha Agrawal | Rahul Ambegaonkar | K Prashanthi | Manoj Rawat |
| Mukesh Bhargav | K Sivadeva Prasad | Nilesh Padhiyar | Rahul S Khubde | K Shyam Sunder | Sanjay Jain |
| Naresh Arya | M V V Gandhi | Nilesh Wani | Rajendra Dhanavai | K Somappa | Sanjay Verma |
| Nemi Chopra | Nambiar Sb | Nimish K Patel | Rajendra Jadhav | Kalyanam Sumar | Tilak Raj Bagga |
| Pratima Kumbhare | S Udaysankar | Pankaj Mistry | Ram Godhane | M A Mushtaq Pasha | V S Issar |
| Pravez Hashmi | V Satya Prasad | Parag Mehta | Ranjana Deshmukh | M Chandra Sekar | Vineet Sabharwal |
| Pravin Kalvit | V Venkata Rao | Parimal Zariwala | Ravi Bhushan | M Nagavendar Rao | Alok Agarwal |
| Rajeshwar Singh | Vijaykumar | Prashant Upadhyay | Ravindra Jhariya | Moinuddin Khan | Jeevan Agarwal |
| Rajkumar Baranwal | Y Mahindra Kishore | Priti Panchal | S N Deshmukh | Muralidhar Lingam | R K Rawal |
| Rupesh Modi | Y Sambasiva Rao Babji | R B Pasi | Sachin Kale | N Paparao | Ruchi Verma |
| Sanjay Bansal | Y Saptanagakumar | Rajeev Wadekar | Sanjay Mahjan | P Devanand | Amrita Ghosh |
| Sanjay Pandey | A Suryalakshmi | Rakesh Jha | Sanjay Nakade | P K Mishra | R K Lalwani |
| Sonal Pagare | B V Satya Narayana | Samira Patel | Sanjay Sanghvi | P Shravan Kumar | Shalini Jaggi |
| Suresh Ranka | Cvs Sastry | Seema Jaisanani | Sanjay Shinde | Pavan Kumar Reddy | Alka |
| Vijay Saxena | D Vijaya Babu | Sheilja Singh | Shailendra P Mahale | Prameela | Sanket Pendsey |
| Visesh Agrawal | K N S S V Chalapathi Rao | Shirish Shah | Shreyans Shah | Rahul Gandhi | Vineet Saboo |
| Vishal Choudhary | K Santhosh Kumar | Urvi Maheshwari | Shrikant Kakad | Ramulu | Yogesh D Varge |
| Ghanshyam Sharma | Mehar N Prasad | Vinod Gidwani | Shrikant Kale | S Sridhar Rao | Zuber Ahmed |
| Jambu Kumar Jain | N B Vijay Kumar | Vipul Maru | Shripad Ratnalikar | Sridhar | A Harwani |
| K G Maheshwari | N Bhavani Prasad | Virendra Shukla | Sunil Sirasikar | Srikanth Reddy | A Kadethankar |
| Kailash Singhal | P S S Srinivas | Yogesh Jain | Swapnil Kendrekar | T Ashok Rao |  |
| Mahesh Goud | S Srinivas | A R Chavan | Swapnil Shah | T Ramu |  |
| Manish Kumbhare | Swapna Sri B | Aarti Ullal | Umesh Aher | T S Chandrashekar |  |
| Manish Sharma | V Ashok | Bhavesh Dhanoria | V Lamkhede | Terala Sreenivas |  |
| Mukesh Dodeja | V V Satyasekhar | Chirag Mehta | V V Wanmali | V K Mansab Dar |  |
| N K Bansal | C Anjani Kumar | Janardhan Bandi | Venkatesh Deshpande | V Laxman |  |
| O P Gurjar | Ch Manoj Kumar | Kinaz Salim Ansari | Vishal Thakare | Venkat Rajaiah |  |
| Pradeep Kothari | D Chenchu Rami Reddy | Mahesh Sharma | Yadav Kudale | Venkat Reddy |  |
| Rajiv Saxena | D Ramesh | Manish Mawani | Amit Achaliya | Venugopal Ramuni |  |
| Rohit Choure | D V S Shoban Kumar | N K Agarwal | Kiran Belsare | Vijaya Mohan Srikonda |  |
| S Chandorkar | G Venu Gopal | Prasad Solanki | Mahesh Gandhi | Anata Sharma |  |
| S K Sengar | I Babu Rao | Rajesh Jiwarajka | Nikhil Patil | B Jaya Raju |  |
| Sanjeev Thareja | K Srinivasa Rao | S P Mathew | Prasad Nikam | Chaitanya Kranthi Kotla |  |
| Shriprakash Singh | K Sudhakar | S S Jain | Rajesh Valvi | D Nagender |  |
| Vidit Khandelwal | L Rama Chandhrao | Sangeeta Beria | Sagar Rakecha | Dilip Nandamuri |  |
| Vinod Sahu | S Rama Krishna | Saurabh Jain | Sarika Deshpande | G Bala Raju |  |
| Virendra Agrawal | S Sudarsan | Shantanu Lokare | Abhay R Wagh | K Hari Babu |  |
| Vivek Vashvani | T V Narayana Rao | Shekhar Shah | Akhil Memon | K Narender |  |
| Yogendra Pradhan | T Venugopal | Suraj Purushottam | Arjun Deshmukh | K S Ashok Kumar |  |
| Yogesh Pandagre | B G V Giridhar | Suresh Kaul | Bhusan Magar | K Uma Maheshwer Rao |  |
| Navneet Agarwal | Ch Srinivasa Rao | Uday V Hegdekar | Chetan Katkade | K Yellappa |  |
| Shachin K Gupta | K Dileep Kumar | Yogesh Velaskar | Dattatray Indurkar | Krishna Mohan Reddy |  |
| Vipin Porwal | Kiranmai Alla | Ritesh M Shah | Debashish Chatterjee | M N Shyamsunder |  |
| Abhishek Shrivastav | Pradeep TVS | Jugal V Gada | Gopal Jawade | M Satya Prakash |  |
| Jaideep Khare | Purna Sreeramaneni | Rahul Wakankar | Karuna Ramteke | Mehmood Ul Haq |  |
| Jitendra Chouhan | S Jayadev | Namita Jiwarajka | Kishor Teple Patil | N Srinivas Rao |  |
| Kalpana Dash | T S Karthik | Sujata Rajwani | L L Bajaj | P Chaitanya |  |
| Vikram Singh Chauhan | Vivekananda | Kunal Doshi | Mahesh Soni | P Raghuramlu |  |
| Anand Lawrence | G D Ratha | Vijay Nandu | Nitin Kallurwar | R Prahalad |  |
| C P Rajesh | I M Jena | SC Gupta | Nitin V Sonone | Shivasubramanyam |  |
| Colin A | J Kishor | A K Saxena | Pradip Parate | Vikram Reddy K |  |
| Ibrahimkutty | Sumit Jhajharia | A K Singh | Pramod Akat | A Raj Kamal Goud |  |
| Kala | Antaryami Sahoo | Ajeet | Rajesh Chavan | Ankit Saxena |  |
| Muraleedharan | B K Mishra | Akhilsh Singh | Rajini Narkhede | Anusha |  |
| N M Arun | J P Das | Arup Das Gupta | Raju Sainani | G Manojlal |  |
| Narayanan Potty | Niroj Kumar Mishra | Ashwani Agarwal | Ravi Waghmare | Geeta Mukka |  |
| Prabhakaran | Paresh Jena | D P Singh | S G Vasista | A Vinaya Sekhar |  |
| Ramachandra Menon | Philips Routray | G C Chitravanshi | S Patankar | Arun Bajaj |  |
| Sajeev Kumar K | R K Goenka | G H Khan | S R Tapadiya | Kumaraswamy |  |
| Shiva Prasanth | S R Samal | I N Basu | Sandip V Gavli | Lilly Rodrigues |  |
| Sreejith K | Samir Mishra | K Srivastva | Sanjay Agrawal | Baba Yunus |  |
| T J L Victor | Srikant Kumar Dhar | Mohd Amir | Santosh Mulewar | C Nithya |  |
| Thomas Paul | Subrat Kumar Behera | P K Tiwari | Santosh Shinde | G Kiran |  |
| Velayudhan | Sudhir Patanaik | P Shukla | Satish Poshattiwar | M Sudhir |  |
| Anil Kumar | Abani Kumar Patro | R B Pathak | Shekhar Godeswar | Kiran Grandhi |  |
| K S Vasu | Ajaya Satapathy | R D Tiwari | Vasant Chaudhary | M A Razak |  |
| N S Sibi | Akash Modi | R S Yadav | Virendra Papalkar | Mohammad Suleman Hussain |  |
| Padmakumar | Asit Behera | Sabir Khan | Yogesh Gosavi | M Raghunath Babu |  |
| Rafeeque Muhammed | Atanu Thakur | Trilok Ranjan | Hemkant M Patil | M Sheetal Kumar |  |
| Shaji Kumar | B K Mishra | W A Ansari | Nilesh Lomte | MD Ajith |  |
| T V Prakash | B N Das | A K Bhatt | Pratibha Pawal | Mohammed Shafee |  |
| V Velayudhan | Biswanath Mishra | A K Chapariya | Shailesh Pitale | Nelakurthy |  |
| Jolly | D N Moharana | Akhilesh Kumar Singh | PARAG PATIL | R Mahendran |  |
| Joy Mathew | Dhananjaya Ram | Anand Singh | SUNIL BHAVSAR | R Rajesh |  |
| K O Joseph | J Narayan Rao | Ankit Chhaparia | UMESH AHER | P G Kamath |  |
| M V Mathew | K B Parida | Dheeraj Singhania | NITIN WADSKAR | P T V Nair |  |
| Neeraj Manikath | Khetra Mohan Tudu | G D Gupta | DILIP DHOPE | Pinkesh Chandra |  |
| P Madhu | M K Chhotray | Monica Gupta | Alok Mathur | Prafull Lolage |  |
| S V Harris | P K Thatoi | R Gupta | G L Dhayal | Prakash Sonarikar |  |
| Satheesh Chandran | Pramod Agarwal | Sunil Misra | G N Saxena | Pramod Gangurde |  |
| Varghese Philip | R K Dalai | V S Goel | I C Mundra | Prashant D Purkar |  |
| Vinod Sebastian | Ratnakar Das | Varesh Nagrath | Prasang Garg | Prashant P Kini |  |
| A R Ajay | S I Ahmed | A K Garg | Shailendra Bhardwaj | Rahul Sathe |  |
| Abdul Gashi | Sanjay Mohanty | D K Gupta | Shailendra Gupta | Rahul Tiwri |  |
| Abraham P George | Santosh Kumar Swain | K K Tripathi | Abhishek Tater | Ravi Utage |  |
| Abraham Varghese | Surjit Sahu | R N Bajpai | Anand Meenawat | Ravindra Ingle |  |
| Anil Vijaya Kumar | B K Das | Rakesh Singh | Anil Samaria | Shaileja Pillai |  |
| Arun | A K Otta | Sandeep Arun | Arvind Gupta | Sunder Krishnan |  |
| Ashok Kumar | A K Singh | Sanjay Singh | Ajay Bansal | Sunit Newale |  |
| B S Jayakumar | A K Sinha | M M Nath | B K Gupta | Umesh Alegaonkar |  |
| Boby V Thambi | Alekh Prasad Mohapatra | P N Gupta | Braj Vallabh Sharma | V V Krishnan |  |
| Francis Mathew | Alok Vijaya Rajkumar Tirky | Rajeev mishra | D C Kumawat | Aditya Mahamankar |  |
| Gopakumar | Amulya Kumar Mishra | Rajesh pandey | Deepak Gupta | Anup Nehete |  |
| Gopalakrishnan D | Arun Kumar Dash | M M Singh | Deepak Sankhla | B S Kaushal |  |
| Jiju Baby Chungath | Biswanath Padhi | Anshul Gupta | G D Ramchandani | Chetan K Mahajan |  |
| Jiju K | Chandan Das | Arun Agarwal | G Devpura | Deepa Reddy |  |
| K P Rajesh | Chinmay Behera | Ashutosh Jain | M N Srivastav | Jagdish Narayan Bedekar |  |
| Abilash Nair | D Garnaik | B S Deval | Manish Goyal | Maithili Lad |  |
| B Jaya Kumar | Girish Behera | H M Rastogi | Mukesh Jain | Sanjay Satale |  |
| Mahesh Sukumaran | Hardip Singh | Mudit Mohan Saxena | Pramod Chourasiya | Sharif Tadvi |  |
| Mini G Pillai | Harihar Praharaj | Rajesh Gupta | Prashant Sharma | Vicky K Dedhia |  |
| Praveen Kumar | Himanshu Nanda | B K Agarwal | Praveen Gupta | Abhijit Pancholi |  |
| Rajiv Philip | J K Dasmohapatra | Ajay Bhargava | Rajeev Narang | Mangesh Paradkar |  |
| Raju A Gopal | J K Mohapatra | Anil Kakar | Ramavtaar Sharma | Mukesh Pednekar |  |
| Sasikumar | K N Dalal | Ankur Upadhyay | Sandeep Jain | Prakash Bhadkamkar |  |
| Tittu Ommen | K P Tripathy | Anuj Garg | Sanjeev Dhuria | Tejal Latiya |  |
| Abilash Chako | Kailash Chandra Das | Anurag Gupta | Sanjeev Garg | B G Baliga |  |
| Ajithkumar | Kamal Dalmia | Arun Kumar Jain | Sanjeev Gupta | Ganpathi |  |
| Anup N | Laltendu Mohanty | Ashutosh Sharma | Shyam Mittal | H Basavana Gowda |  |
| C M Peter | M F Alam | Atish Sharma | Suresh Medatwal | K M Srinath |  |
| Dennis Varghese Thomas | M N Das | Avinash Kr Singh | Ajay Singh Rathore | Karunesh Kumar |  |
| Harish Kumar | Madan Mohan Samal | Avnish Kumar Jain | Dinesh Sharma | M V Krishna |  |
| Johny Cherian | N M Sahu | B B Maheswari | Gopal Khandelwal | Sanjay Rao |  |
| Jose Ukken | P C Bahinepati | B K Gupta | Mahesh Gupta | V Shankar |  |
| Josemon Thomas | P C Mohanty | D B Gautam | Rajendra Prasad | Arun Padaki |  |
| Joseph Philips | P K Dash | D K Goel | Ramesh Gupta | Sanjeev Rao Girimaji |  |
| Kannan T | P K Majhi | Iqbal Ahmad | Swati Srivastav | Shashidhar |  |
| Madhu Kumar | P K Panda | M Naved | T P Sharma | Chaithanya Murthy |  |
| Mohan Thomas | P K Sahoo | Naveen Jameja | Abdul Gafar | Chandan Kumar H N |  |
| Mujeeb Rahman | P Sivram | P K Gupta | Amolak Golcha | Chandrika K M |  |
| Nelson | P Syam Sundar | Rahul Mittal | Anil Khurana | P J Rahul |  |
| P R Rajeev | Prafulla Kumar Dash | Rajeev Kumar Gupta | Arun Agarwal | Rajanikanth B |  |
| P Shaji | Pranay Kumar Patro | Rajesh Aggarwal | Balveer Choudhary | Ramachandra Prabhu |  |
| Prethweerajan K | R K Mohapatro | Ravinder Singh | Kapil Gupta | Ramesh R K |  |
| Radhakrishna Pillai | R K Sethi | S K Gupta | M K Bharti | Reena Mathew |  |
| Sai Lal M | Rabindra Ku Das | Vikas Agarwal | Mahesh Mavalia | Renuka Prasad A R |  |
| Shanto | Ramakanta Panda | Vikas Jain | Mukesh Gupta | Reshmi |  |
| Sreedharan | S K Nayak | Vinay Ahoja | Pawan Bhardwaj | Sridhar Vaidya |  |
| Thampy Varghese | S Kabi | Amit Maheswari | R K Jain | V Prasanna |  |
| Thomas Sebastion | S Rajkumar | M K Bansal | Rajesh Agarwal | A N Ramesh |  |
| Tomy | S S Agarwalla | Mohit Tandan | Rajesh Jain | Amarnath K A |  |
| Veeran Muhammed | Sameer Panda | Mukesh Agarwal | Suresh Gupta | Anil Kumar H |  |
| Abubaker Zeeshan | Satyanarayan Rao | Nikhil Pursnani | T C Mahawar | Aswathy Mary James |  |
| Mohiyudheen Hijas Abbas | Seema Roy | Rakesh Mittal | Vivek Mehta | B V Pradeep |  |
| Sasi Kumar | Siba Prasad Dalei | S K Rathi | Yogesh Choudhary | Dayananda G |  |
| Kripal M K | Sudhanshu Sekhar Sethi | Suyash Sharma | Abhinav Kumar Gupta | Diwakar |  |
| Mahesh G Thampi | Susanta Kumar Panigrahi | V K Bindra | Abhishek Hajela | Girish M |  |
| Mathew Jacob | Tapan Nayak | Vinay Kumar Gupta | Ajay Shah | Govindappa |  |
| Moideenkutty Gurukal | Trinath Panda | A K Jain | Atul Dhingra | Hansraj Alva |  |
| N K Unnikrishnan | Tushar Kanti Sahu | Absar Ahmad | Balram Sharma | Haraprasad L |  |
| Nishad | U K Mishra | Amit Sharma | Deepak Gupta | Harish |  |
| P Athmaram | Uma Mishra | B K Gupta | Hardev Nehra | Hrs Murthy |  |
| P Naseer | V V L N Rao | Lokesh Kumar | Hema Singh | K Manjunath |  |
| P Vijayakumar | Ankita Tiwari | Param Veer Chauhan | Mona Dhingra | Kabadi |  |
| P Vinodkumar | Debarchana Jena | Puneet Bhaseen | Mukul Gupta | Kiran J |  |
| Pe Moosa | Debasish Patro | Vijay Maithani | Prem Prakash Patidar | M Bhanukumar |  |
| Pradeep Sreedhar | Ipsita Mishra | Pankaj Aggrawal | Rajeev Kasliwal | M G Pradyumna |  |
| Prasanthakumar | Jayashree Swain | Yogesh Yadav | Ravindra Kumar Shukla | M Manjunath |  |
| Prathapachandran | P K Mishra | Mukhlesh Gupta | Sailesh Lodha | Manjunath M |  |
| Praveen | Subash Ranjana Behera | Vivek Agarwal | Sanjay Saran | Mohammaed Ismail |  |
| Rajalakshmi | Swayamsidha Mangaraj | A I Hanfi | A Syed Abuthaahir | Muneer Ahmed |  |
| Rajendran | G Shivkumar | G S Sehgal | Ahamed Naina | P M Patil |  |
| Ramachandran | Gokulnath Premchand K S | Mohammad Fahad Waseem | Ajith Prasad | Pankaj Singhai |  |
| Sameer Babu | J P Johnson | Nandini Rastogi | Anbu Selvam | Prakash N |  |
| Shaji | Jason Jerold | R P Shukla | Annamalai | R S Raju |  |
| Shibin T Sudevan | K R Ramanathan | Sakshi Manchanda | Ashok Kumar | Ramesh |  |
| Sivakumar M | Muruganatham | Siddharth Madnani | Bala Krishnan | Rangaswamy |  |
| Sooraj | P G Sankaranarayanan | Subodh Jain | Benedict Aruldas | S F Itaraj |  |
| Sunny John | P Rakesh | A K Verma | E Arumugam | S S Raju |  |
| Suresh Kumar P | R Karthikeyan | Anubha Srivastwa | K A Venkatachalam | Sandeesh Rai |  |
| Zakeer N P | R Sharmila | Anurag Agarwal | K Sivakumar | Sayed Mohsin |  |
| A A Pangi | T S Prabhuram | Ashok Solanki | M Arunachalam | Sheetal Kamath |  |
| A R Devareddy | P Krishnamurthy | Asutosh Bajpai | M Manoharan | Shiva Kumar N S |  |
| Gopal Naik | P L Saravanan | Atul Mathur | N Nagajothi | Shobanaidu |  |
| Khwaja Naseeruddin | R Rajarathinam | B P Agrawal | Nambi | Sunil R |  |
| Manikappa S | R Viswanathan | Bhupesh Papney | P Arunkumaran | V Prabhakar |  |
| Rajshekhar Patil | Ravi Shankar | Harish Srivastava | P Chowdappa | Venugopal |  |
| S B Hegde | S Senthil Kumar | J S Kushwaha | P M Karthigayan | Vijay Kumar |  |
| Sachin Gudage | S Sethuraman | Manas Tondon | P Saravanan | Y N Venktesh |  |
| Venkatesh Desai | Suresh Kumar | Manmeet Singh | Pradeeshkumar | Y S Ravi Kumar |  |
| Vijay Naik | K Mohan | R Dayal | Prakesh Kennedy | A N Srinivas |  |
| Vijay S Maitri | K Vivek Narayanan | R K Sharma | R Kannan | Amalaselvam A |  |
| Nithish Shanbhag | Karthik | R P N Singh | R Rajkumar | C S Ravindra |  |
| Vishwanath C Naragond | Kavitha | R S Gupta | Ram Mohan | Chikkalingaiah |  |
| Ajith Kulkarni | S Mathanagopal | Rajesh Srivastwa | Ramesh Sadhasivam | Dhamodhar |  |
| Ameet Khatawakar | T K Senthil Kumar | Ramashrya Singh | S A Kabeer | Dinesh V Kamath |  |
| Anand Koppad | T Sugumar | S K Gupta | S Geetha Thiruppathi | K Mohan Kumar |  |
| Arvind Yalamali | K H Salim | S P Singh | S T Ramachandran | Kishore N L |  |
| Basavaraj Patil Raikod | A Joseph Panneerselvam | Sanjay Singh | S Vijay Alagappan | L S Adarsha |  |
| D Y Loyalekar | B Muruga Prakash | Satyendra K Sonker | Sairamanan | Nitesh P |  |
| Dhanpal Hegade | Dilip Kumar | Shivanjali Kumar | Sanjee Vasu Dhavan | Sanjay S |  |
| G H Shivayogi | G K S Sudhager | Sudeep Sarkar | U Subash Rau | Shiva Kumar B R |  |
| Gopi | G Sriram | V B Srivastava | V S Rajkumar | Sudindra |  |
| M I Shaikh | K Arun Prasad | V K Vermani | V T Bhaskaran | Younus Saleem M H |  |
| Mahendra Kare | Latha Ramalingam | Veerendra Singh | Vasanth | A S Sanjaya |  |
| Manoj Pujar | M R Swaminathan | Y K Arora | Vasu Mathi | Abdul Mateen Athar |  |
| N P S Savaikar | Pandian S | Abhay Km Srivastava | Venkatesan | Ananth Shenoy |  |
| Nitin Agarwal | Paul Sudhakar M | Ajay Katiyar | Victor | Bhaskar Palan |  |
| Pradeep T V | R Muthukumaran | Arun Sharma | Vijayalakshmi | Chethan Abraham |  |
| Praveen Bhadre | R MUTHULAKSHMI | Atul Kharbanda | B Ananda Kumar | G Prakash |  |
| Praveen Kusubi | S Aravind Raj | Atul Srivastava | J Prabakaran | H R Prassana Kumar |  |
| Rajendra Parak | S Sadhiq Ali | Dinesh Kumar | Kalaivanan | Hm Kumar |  |
| Rajesh Naik | Satheesh Vincent S | G S Sinha | Mohan Kumar | K M Sridhar |  |
| Ravi Kumar | V Ramasamy | Gopal Gupta | P S Prashant | K Nagesh |  |
| Ravindra B M | P Jegan | Indevar Sharma | P Selvakumar | M N Raju |  |
| Sachin Bongle | R Arulprakash | Kamlesh K Gupta | Rajeshkumar | Raghuram |  |
| Suresh S R | S Vijaya Bhaskar Reddy | Monica Khanna | Srinivasa Kannan | Rajaram |  |
| U R Raju | M Thakur | Piyush Dixit | Su Bhalaji | Raveesh R |  |
| Uday Bande | Niranjan Kumar | Rahul Kapur | Sunil Singhvi | Shankar Prasad |  |
| Vikas M Punith | Anand Shankar | Rajeev Agarwal | G Thirumoorthi | Shekar Babu |  |
| B E Kalinga | Atul Kumar | Ramesh Arora | A Soukat Ali | Shivakumar |  |
| B M Vishwanath | K P Lal | S Najimahammad | K Jayaraman | R N Jain |  |
| E M Surendra | Praveen Shankar | Sameer Tondon | Lakshmipathy Ramesh | Harsh Durgia |  |
| G B Sattur | V K Dhandhania | Vishal Chopra | M Manjula Ganesh | Parag Shah |  |
| G S Mahishale | Awadhesh Kumar | Vishal Kumar Gupta | P Balamanikandan | Pradip Dalvadi |  |
| Kasal Srinath | B Mondal | Y C Sharma | S G D Gangadharan | Ramesh Goyal |  |
| Prakash Ramanagoudar | B N Prasad | Abhishek Agarwal | Sridevi Anantharaman | Shrikant Somani |  |
| R N Belagaonkar | B P Chamariya | Anand Bajpai | A Prabhu | Vivek Arya |  |
| Rajeshwar Naik | Chandan Kumar | Arvind Singh | A Sethuramashankaran | Vivek Patel |  |
| Sachin Hosakatti | D Kumar | D Dev Roy | B V Mohan Sundar | Shashank Zalak |  |
| Sha Abrar | Gopal Baran Chattopadhyay | Devesh Rajani | C Jayapal | Aditya S Bari |  |
| Shashidhar | Madan Prasad | H S Sodhi | D Dhanamithiran | Balaji Puri |  |
| Shivanand Nellogal | Maj P K Sinha | J K Yadav | Jaswanth Khatod | Bharat Asaram Salve |  |
| Shivashankar | Md D Shamim | Jitendra Shukla | K Baraneedharan | Bhaskar Patil |  |
| Arun Patil | Navin Kumar | K P Chandra | K Mohan | Dnyanoba K Bhaskar |  |
| Deepak Deshpande | O P Shah | P K Singhaniya | K Senthil Kumar | Mahendra M Deshmane |  |
| Digambar Naik | R K Keshab | Prem Prakash | K Sumathi | Nilesh Kulkarni |  |
| Gopal D | R P Chaudary | R K Singh | K V Sathyanarayan Sa | A D Kulkarni |  |
| Krupadevi | Rajiv Lochan | Rajat Dixit | Kalaiselvi Vairavel | Abhijit Vaidya |  |
| M S Kadapatti | S K Sinha | Rajesh Gupta | L Meenakshi Sundaram | Ajit Birnale |  |
| Madhav Prabhu | S P Srivastawa | Rajesh Nath Pandey | M Jawahar | Amit Palange |  |
| Manoj Varakeri | Sachchidanand Prasad | Rohit Choubey | M N Noorjahan Beevi | Amol Karpe |  |
| Moin Sabeer | Sandeep Kumar | Sailash Srivastava | M Rajkumar | Aniruddh S Phadake |  |
| Nagbhushan | Sanjay Kumar | Shailesh Dwivedi | P Boopathirajan | Aniruddha Surendra Umrani |  |
| P K Pai | Shambhu Kumar | Vipul Agarwal | P Selvapaandian | Arati Kapale |  |
| P Ramamurthy | U K Benerjee | Anurag Bajpayee | R Kamalakannan | Asha S Babar |  |
| Patrick | Ajay Kr | Arun Pandey | Ramesh Chandrasekaran | Asmit Vaidya |  |
| Prakash Nayak | Arun Kumar Sinha | Maneesh Gutch | Roopesh Jain | Avinash Panbude |  |
| R R Walwekar | Arvind Kumar | Santosh Chaubey | S Krishnan | Bharat Jain |  |
| Rajeev A Malipatil | B K Thakur | Alpa C Paneliya | S P Prabhakar | Bharati |  |
| Rajesh Sheat | D P Singh | Birju Mori | Shaik Sulaiman Meeran | C P Patil |  |
| S M Biradar | O P Saha | Chirag V Aghara | Srivatsa | Chidanand Aawlekar |  |
| S V Konnur | V K Pandey | Dishank Patel | Suresh | Danish Memon |  |
| Sangram Biradar | P N Singh | Kalpesh S Patel | T S Gopinath | G Siddhapure |  |
| Santosh Vastrad | Pawan Mehta | M A Karmur | Thanakeerthi | Harshal Patil |  |
| Satish | R K Mishra | Vitrag Shah | U V Mohan | Jabbar V Desai |  |
| Satish B Patil | Shyam Kumar Shroff | Yash Patel | Uma Mahesh | Jagannath Dhadwad |  |
| Shivamurthy T | Vinay Kumar | Alok Shah | V Ganapathi Krishnan | Jayant Mannikar |  |
| Shivanand Bodihal | Neeraj Sinha | Apoorva Madia | V Padma | Kapil Patil |  |
| Sowmya | A Kenish | B M Shah | V Rajendran | L S Kabra |  |
| Sreenivasa Nayak | A Omar Faroque | Bharat Vanani | V Sanjay Moses | Madhuri M Shingade |  |
| Swati Y Kamshetty | G Jagadeswaran | Bhavesh Patel | Vikram Vasagan | Manasi V Harale |  |
| Tyagaraj | Moorthy | Bhavesh Patel | G Shamugasundar | Manisha Deshmukh |  |
| Vijay Desai | R Gurusamy | Bhavin Fadadu | R Ramkumar | N M Beke |  |
| Preetham | Ravikumar | Bipin Patel | T S Boochandran | Nilesh Bhokre |  |
| Raveesh | Revathi Harinaran | Danish Kanpurwala | Ajay Gupta | Nishikant Maske |  |
| Sandhya Kulkarni | S P Hemanand | Dhiren Joshi | Munish Prabhakar | Nitin Gawade |  |
| Anil Satyaraddi | Senthil Kumar | Dinkar Goswami | Subhash Tyagi | Nitin Lohakare |  |
| Manjunath Ankal | Shanmugasundram | Hiren R Shah | Umesh Verma | P K Singhal |  |
| Manjunath Goroshi | Sr Anbalagan | Hiten Barot | Vivek Jha | P R Joshi |  |
| Praveenkumar Devarbhavi | Sujith Kumar | I C Mundra | Parul Khurana | P V Joshi |  |
| Ravikumar V Ryakha | Suresh Kumar | Ilesh Mehta | A K Malhotra | Pankaj Nilapwar |  |
| Sandeep Donagaon | Varatharajan | Jaimin Shah | A K Taneja | Prashant Chole |  |
| Vaibhav Vasudev Dukle | Arul | K Doshi | Ankit Gupta | R R Gundeli |  |
| Varunchandra Alur | G Jayaprakash | Ketan Chokshi | Anuj Mittal | Rahul Doshi |  |
| Amit Kumar Kalwar | Kumar N | Keyur Thakor | Ashwani Nagpal | Rahul Gadekar |  |
| Apurva Parekh | M Madhavan | Kishan Jani | Jaya Jain | Rahul Ware |  |
| B P Chakraborty | Raja | Kishor Viradiya | Lkc Sinha | Ravindra P Chhajed |  |
| D J Paul | T Saravanan | M P Patel | P K Babbar | Ravindra Satalkar |  |
| Dwijen Das | Anwar Ali | Milin Patel | Prateek Bajaj | Rohan Kate |  |
| Kabir Dutta | Cheralathan | Niranjan Chodhary | R K Singh | S N Kore |  |
| Kamal Nahata | G Sugumaran | P C Trivedi | Rajeev Agrawal | S P Jategaokar |  |
| Kaushik Dutta | Muralidharan | Prakash Kurmi | Ravinder Kumar | Sanjay Katke |  |
| Mridul Bera | P Arthi | Raxit Brahmbhatt | Rohit Goel | Sanjay Thorat |  |
| Naveen Agarwal | P Chandrasekar | Rumin Shah | Rohit Jain | Satish Godse |  |
| Nitya Gogoi | P S Somasundram | Rushiraj Suthar | Romil Chhura | Satish Guthe |  |
| Pranabes Ray | R Srinivasan | Safi N Shaikh | Shashank V Kumar | Shagupta Bagwan |  |
| Rama Prasad Medhi | S Sureshkanna | Sandeep Patel | V P Mehta | Shailendra D Mane |  |
| Soupayan Dutta | Sai Devi | Sandip Shah | Vandana Garg | Sharad Kamble |  |
| Subrata Kundu | Sasi Rekha | Suresh Damor | Vishal Aryan | Shital Gosavi |  |
| Sumantra Mukhopadhaya | Vijay Giri | Vashant Mungra | Yogesh Kautish | Shounak Annachatre |  |
| Amitabha Roy | C Arunpradeep Raja | Vinay Patel | Amit Jindal | Shrikant Kasar |  |
| S Kothari | V Dineshkumar | Vinod Pathiria | Arun Moondhara | Sushma Jadhav |  |
| A Ahad | A M Karthik Kumar | Vipur Vekariya | Ashok Chandna | V B Purandare |  |
| A Sharma | Chidambaran Gopu | Yayati Dave | Atul Gupta | Vaibhav Lotake |  |
| Apurba Banerjee | Jothi Ramalingam | Advait N Dholakia | Deepti Gupta | Vaishali Pathak |  |
| Arunava Dasgupta | K Suresh Kumar | Alpesh Patel | Dinesh Gupta | Vasudha Sardesai |  |
| Ashok Kumar Roy | Kumaresan | Anil Undhad | Kushal Mathur | Vijay Amritkar |  |
| Avijit Ganguly | M Loganathan | Anish Mansuri | M Mehra | Yogesh Aher |  |
| B C Bhagawati | N Marichamy | B S Shah | Pankaj Kumar | Yogya Jha |  |
| Bimalendu Chattapadhyay | P Govindaraj | Bharat Kela | R Ganesh Kumar | Deepak Salunke |  |
| Debdas Chakraborty | P Thandavan | Darshan Shah | Rajiv Gupta | Dhanshri Tayshete |  |
| G Poufullong Kabui | Pratheba Nanthakumar | Deepak P Munshi | Ramanabhi | Girish Borawake |  |
| Indrajit Paul | Ramesh Raja Prabhoo | Dipak R Bhadiyadara | Samant Jain | Jitendra Agarwal |  |
| Jayanta Talukdar | S Satish Kumar | G R Badlani | Sandeep Sahay | Mahendra Kawediya |  |
| Kalyan Mitra | Senthilkumar | Gaurav Chaya | U N Shahi | Mahesh Jadhav |  |
| Kausik Munsi | Senthilkumar S K | Ghanshyam V Patel | Vkg Nair | Narendra Javdekar |  |
| Kh Lokeshwar Singh | Sivaprakasam | Girish Pokharna | D S Mathur | Rajesh Patil |  |
| Krishnendu Dey | Subashini Devi | Gopal Shah | P R Aryan | Ramesh D Kurle |  |
| M Ratankumar Singh | Suresh Kumar | Hardik Suthar | Sanjay Dhall | Ravindra Ghongade |  |
| Mahabendra Nayak | T R Gnanasambandam | Jagdish B Patel | Sunil Massand | Sachin Yadav |  |
| Mamtaz Ahmed | Thennarasu | Johnson Samuel | Vinod Kumar | Sanjay Gandhi |  |
| N G Goswami | Udhaya N S | Juzoor Limdiwala | Sunil Mishra | Sanjeev Akherkar |  |
| N R Das | Premkumar | Kamlesh Phatania | Dinesh Dada | Shaliwahan Pattanshetti |  |
| P P Chetia | Sathian Raghavan | Mahesh R Vasanie | Amit Moudgil | Shrikant S Wasvade |  |
| Paramartho Bhattacharya | Abhijit Jadhav | Mahesh Sanghavi | Anju Batra | Vidya Patil |  |
| Ranjit Dey | Benny Negalur | Mehul Shah | Ashish Sexsena | Yash Bahulikar |  |
| S Ibomcha Singh | Jaydeep Shinde | Mukul Oza | Ashok Saraf | Ganeshkumar S Satpute |  |
| S K Nasirudin | Jenish J Vira | P K Gandhi | Ashwani Mittal | Gjanan Gondhali |  |
| S M Baruah | Kaustubh Durve | Paritosh Mehta | Davinder Gupta | Kiran Salunkhe |  |
| Saibal Adhikary | Manish D Dhadse | Piyush Patel | Deepak | Mahesh Kagli |  |
| Saibal Chakraborty | Milind A Kadam | Pradip Patel | Gurpreet Bawa | Manoj D Devhare |  |
| Somnath Roy | Nikhil D Prabhu | R H Patel | H S Makhan | Mulay Sanket Dattatraya |  |
| Sourav Biswas | Priti Sanghavi | R P Padiya | Munish Khurana | Nitinrane Kavitkar |  |
| Subhasis Mukherjee | Rohini S Gajare | Rajesh Trivedi | Nitin Mittal | Prafulla Wargane |  |
| Subrata Bhattachriya Vcm | Rukiya H Surya | Rakesh Koya | R P Gaba | Vasant Karmarkar |  |
| Subrata Kumar Pal | Rupali N Bhoye | S T Rangoonwala | Ravinder Singh | Vineet Rao |  |
| Sudipta Roy | Vinod Mehtil | Sandip Desai | Rohit Khurana | Vinod Awhad |  |
| Sujay Kumar Mukhopadhyay | Ajit Vora | Sanjay Chaudhry | Sandeep Chabra | Vyanktesh Patki |  |
| Suman Chakroborty | Amit Shah | Saurin Upadhyay | Sandeep Chaudhary | Mohan Magdum |  |
| Sunanda Adhikari | Anil Parmar | Shabbir Nandarwala | Sumesh Khanna | Suhas Khaire |  |
| Supratik Chakraborty | Arun Shinde | V S Patel | T S Didwal | Chetan Patil |  |
| Apurba Mukherjee | Devendra Dhopte | Vishal Shah | Tejinder Sikri | Harshwardhan Bora |  |
| Sushil Kumar Das | G S Kundi | A I Maljiwala | Vikas Kaushal | Rupesh Nagrale |  |
| T Jeetenkumar | Gaurav Sahashrabuddhe | B B Sing | Zia Ah Qazi | Rajesh Munde |  |
| T K Chattaraj | Harish Thakkar | Chirayu Vaidya | Alok Gupta | Ajay Rotte |  |
| Tamanash Bhattacharya | J L Daga | Dhaval Sheth | Charanjit Singh | Ashish Sunde |  |
| Bhargav Nath | M Kokate | Hardas Patel | J S Pannu | Deepak Bhosale |  |
| Tarun Chakraborty | Manish Itolikar R3 | Jinen M Shah | Jasjit Singh | Madhuri Kirloskar |  |
| Th Premchand Singh | Manish Pendse | Kiran Patel | Jasminder Singh | Pranjali Wanmali |  |
| Tushar Kanti Batabyal | Mehak Raj Advani | Nilesh Bhatt | Kursheed Qureshi | R B Sharma |  |
| Udas Ghosh | Neeraj Tulara | Parth B Patel | Makhan Lal Gupta | Rameshwar Palsikar |  |
