# Supplementary material for: Cardiovascular risk in newly diagnosed type 2 diabetes patients in India
Source: PLoS One. 2022 Mar 31;17(3):e0263619. doi: 10.1371/journal.pone.0263619 (PMC8970505; doi:10.1371/journal.pone.0263619)
Supplement: S2 File — (PDF) [file pone.0263619.s003.pdf]

India Diabetes Study (IDS)

Eris

TABLE OF CONTENTS

	CONTENT	PAGE NO.
	**INVESTIGATOR APPROVAL**	**1**
	**ABBREVIATIONS AND DEFINITIONS OF TERMS**	**2**
	**ABSTRACT**	**3**
	**OBSERVATION PLAN SYNOPSIS**	**4**
**1**	**BACKGROUND INFORMATION AND NEED FOR STUDY**	**6**
1.1	INTRODUCTION	6
1.2	NEED FOR STUDY	6
**2**	**STUDY OBJECTIVES**	**6**
2.1	PRIMARY OBJECTIVE (OR AIM)	6
**3**	**METHODOLOGY**	**7**
**4**	**CONSENT OF THE INVESTIGATOR**	**8**
**5**	**INCLUSION & EXCLUSION CRITERIA**	**8**
5.1	INCLUSION CRITERIA	8
5.2	EXCLUSION CRITERIA	8
**6**	**STUDY PROCEDURE**	**9**
6.1	SCREENING VISIT	9
**7**	**STUDY EVALUATIONS AND MEASUREMENTS**	**9**
7.1	BASELINE PARAMETER	9
7.1	LABORATORY INVESTIGATION	9
7.1	SCREENING AND MONITORING EVALUATIONS	9
7.1	LABORATORY EVALUATIONS	9
**8**	**STATISTICAL CONSIDERATIONS**	**9**
8.1	STATISTICAL METHODS	9
8.2	CLINICAL ADVERSE EVENTS	9
8.3	DEFINITION OF A SERIOUS ADVERSE EVENT (SAE)	10
**9**	**STUDY ADMINISTRATION**	**10**
9.1	DATA COLLECTION AND MANAGEMENT	10
9.2	DATA SOURCES (IF APPLICABLE, FOR EXISTING RECORDS)	10
9.3	CONFIDENTIALITY	11
**10**	**REGULATORY AND ETHICAL CONSIDERATIONS**	**11**
10.1	DATA SAFETY MONITORING PLAN	11
10.2	RISK ASSESSMENT	11
10.2.1	POTENTIAL BENEFITS OF STUDY PARTICIPATION	11
10.2.2	RISK-BENEFIT ASSESSMENT	11
10.3	RECRUITMENT STRATEGY	11
10.4	INFORMED CONSENT	11
10.5	PAYMENT TO SUBJECTS/ FAMILIES	12
	**REFERENCE**	**12**
	**CASE REPORT FORM**	**13**
	**SUBJECT INFORMATION SHEET AND INFORMED CONSENT FORM**	**113**

"A cross-sectional (baseline) & observational real world study on newly diagnosed patients with type 2 diabetes in India."

OBSERVATION PLAN

India Diabetes Study (IDS)

Study Short Title	India Diabetes Study (IDS)
Study No.	ERIS/OS/20/001
Version No.	01
Date	Apr 23, 2020

CONFIDENTIALITY STATEMENT

The information provided in this document is confidential. It is provided to you as a co-principal investigator for review by you, your staff, and applicable institutional ethics committee. By accepting this document, you agree that the information contained herein will be kept confidential, unless required by government regulations or laws.

OBSERVATION PLAN

Title: A cross-sectional (baseline) & observational real world study on newly diagnosed patients with type 2 diabetes in India

Short Title: India Diabetes Study (IDS)

Study Principal Investigator: **Dr. A. G. Unnikrishnan**

Site Code: | | | | | | | |

Name of Investigator: ______________________________

Date: ______________________ **Sign & Stamp:** ______________________

CONFIDENTIALITY STATEMENT

The information provided in this document is confidential. It is provided to you as a co-principal investigator for review by you, your staff, and applicable institutional ethics committee. By accepting this document, you agree that the information contained herein will be kept confidential, unless required by government regulations or laws.

ABBREVIATIONS AND DEFINITIONS OF TERMS

BMI	–	Body Mass Index
CDSCO	–	Central Drugs Standard Control Organization
CGMS	–	Continuous Glucose Monitoring System
CRF	–	Case Report Form
GCP	–	Good Clinical Practice
HOMA-IR:		Homeostasis Model Assessment-Insulin Resistance
ICH	–	International Council of Harmonisation
ICMR	–	Indian Council of Medical Research
IDI	–	Insulin Disposition Index
LDL	–	Low Density Lipoprotein
MNT	–	Medical Nutrition Therapy
MOHFW	–	Ministry of Health and Family Welfare
SAE	–	Serious Adverse Event
SOC	–	Standard of Care
SPSS	–	Statistical Package for Social Sciences
WMA	–	World Medical Association

ABSTRACT

Context: (Background)

According to the latest IDF 2019 data approximately 463 million adults (20-79 years) were living with diabetes and by 2045 this will rise to 700 million. The proportion of people with type 2 diabetes is increasing in most countries. In total 79% of adults with diabetes was living in low and middle income countries.

India is home to more than 77 million people with DM and is estimated to have the second highest number of cases of DM in the world after China, as per the IDF Atlas 2019.

Over the past few decades, various studies have been done to attempt to estimate the prevalence of diabetes in India. But till now no study have been done to characterize and classify newly detected type 2 diabetic population.

Objectives:

To characterize/type/profile of the newly diagnosed Indian patient with diabetes from demographic details and laboratory investigation.

Setting/Participants:

The purpose of this study is to include 1300 investigators @ 10 subjects/participants per investigator

Study Interventions and Measures:

Subject's baseline socio-demographic, anthropometric data will be recorded and the subject's routine laboratory variables will be analyzed in a centralized lab.

OBSERVATION PLAN SYNOPSIS

**Study Title**	A cross-sectional (baseline) & observational real world study on newly diagnosed patients with type 2 diabetes in India
**Study Short Title**	India Diabetes Study (IDS)
**Study Rationale**	• Worldwide the burden of diabetes increasing tremendously. India ranks 2nd after China having the highest number of diabetic patients. According to recently published IDF Atlas 2019, 9th edition, 77 million Indians are suffering from diabetes amongst 643 across the world. 1 in 6 adults with diabetes in the world come from India.<br>• The overall prevalence of diabetes in the country is 11.8 percent, with men and women almost equally affected. As many as 60.5 percent of India's diabetes patients lack blood sugar control despite 85.7% receiving oral medication to manage their condition. Even the incidence of silent hyperglycemia in a newly detected type 2 diabetes patient can be present years prior to diagnosis, leading to the existence of microvascular and macrovascular complications even at the time of initial presentation. It is estimated that up to a third of people with diabetes are undiagnosed, with the hypothesized lag time between its onset and diagnosis averaging 4 years to 7 years. Even diabetes complications are progressive and it leads to significant morbidity and mortality.<br>• Against this background an understanding of the changing epidemiology of diabetes in India is required. There have been studies which are done to estimate the prevalence of diabetes in India but no such studies have been done to characterise and classify newly detected diabetes population.
**Study Objective**	To characterize/type/profile the newly diagnosed Indian patient with type 2 diabetes from the demographic details and baseline investigations
**Inclusion Criteria**	**Patients will be entered into this study only if they meet all of the following criteria:**<br>• Adult patients (Aged 18 Years or above) who were newly diagnosed with type 2 diabetes and referred to the clinic within 1 year of diagnosis.<br>• Diagnosis of diabetes will be based on American Diabetes Association criteria.
**Exclusion Criteria**	**Patients that meet any of the following criteria must not be enrolled in the study:**<br>• Monogenic diabetes syndrome<br>• Disease of the exocrine pancreas<br>• Gestational Diabetes<br>• Acute or severe chronic heart disease<br>• Renal or psychiatric disease

**Number Of Subjects**	The study will include 10 participants and 1300 investigators
**Study Duration**	The study will start by June 2020 and it is expected to be completed by March 2021
**Data and Safety Monitoring Plan**	Because of the observational design of the study the subject will remain under control of their treating physician. All the data will be directly transferred to a computer to prevent registration errors.<br><br>Data quality management will be the responsibility of Eris Lifesciences Limited. The principal investigator will have access to all data during the study. There will be 33 regional coordinators who will have access to the data from all subjects in their respective region. The 1300 participating doctors will have access to the data of their own subjects

1. BACKGROUND INFORMATION & NEED FOR STUDY

1.1 Introduction

Worldwide the burden of diabetes increasing tremendously. India ranks 2nd after China having the highest number of diabetic patients. According to recently published IDF Atlas 2019, 9th edition, 77 million Indians are suffering from diabetes amongst 643 million across the world. 1 in 6 adults with diabetes in the world come from India.

The prevalence of diabetes in India is extremely high and continuing to rise rapidly. Asian Indian phenotype, higher insulin resistance and hyperinsulinemia, low adiponectin levels, high carbohydrate diet (always high PPG), visceral adiposity (thin fat Indian), late diagnosis, Indian incretin axis, sympathetic over-activity are important factors responsible for the higher prevalence of diabetes (and pre-diabetes) in India.

The purpose of this study is to capture the baseline data of newly diagnosed type 2 diabetes patients and characterizes the patient population according to concomitant risk factor stratification, concomitant clinical conditions/co-morbidities and complications (microvascular, macrovascular) and followed up for at least one year. This study will be a one its kind real world study and will give meaningful insights for future diabetes management in India.

Incidences of silent hyperglycemia can be present for years prior to diagnosis, leading to the existence of microvascular and macrovascular complications at the initial presentation. It is estimated that up to a third of people with diabetes are undiagnosed with the hypothesized lag time between its onset and diagnosis averaging 4 to 7 years. Even diabetes complications are progressive and lead to significant morbidity and mortality. Similarly, NAFLD is frequently associated with diabetes and has emerged as a major risk factor for end-stage liver disease and is a predictor of cardiovascular disease. Ditto, obstructive sleep apnea with which NAFLD frequently co-exists.

Systematic classification of diabetes is important and has implications for treatment strategies. It is not an easy task and many patients do not fit into a single class, especially younger adults, and 10% of those initially classified may require follow ups.

The prospective observational multicenter India Diabetes Study (IDS) will monitor the natural course of disease, from first year after diagnosis, with focus on comorbidities and complications, using comprehensive phenotyping. India Diabetes Study will be a one of its kind large scale real world study which will be focused on private practitioners and newly diagnosed Indian patients with diabetes to understand the baseline characteristics, concomitant risk factors and clinical conditions, diagnosis and treatment.

1.2 NEED FOR STUDY:

- Over the past few decades, various studies have been done to estimate the prevalence of diabetes in India. But till now no study has been done to characterise and classify the newly detected type 2 diabetic patients
- No single large scale study has been focussed on the private practitioners and newly detected diabetes patients to understand the baseline characteristics, concomitant risk stratification, clinical conditions, diagnosis and treatment, in the real world.

2. STUDY OBJECTIVES

2.1 Primary Objective (or Aim)

- To characterise/type/profile the newly diagnosed Indian patient with type 2 diabetes from the demographic details and laboratory investigations

3. METHODOLOGY

3.1 Study Design:

It will be a cross-sectional observational study among private practitioners. In the study, subjects/participants have to be newly diagnosed cases of type 2 diabetes mellitus (diagnosed within the last 6 months to a year). After satisfying the case definition and obtaining informed consent, newly diagnosed type 2 DM subjects/participants will be enrolled. After explaining the details of the study, a comprehensive case history will be recorded in a case report form, per routine clinical practice. The baseline details will include data on socio-demographic details, family history details, and medication details such as age, sex, educational status, economic status, smoking and tobacco chewing status, alcohol consumption, diet and physical activity. All subjects will be interviewed regarding family history, medical history and other comorbidities the way it happens in usual clinical practice.

The selected group of subjects will be sent for routine laboratory investigation in a centralized laboratory (SRL) which will include blood tests such as the fasting plasma glucose, post-prandial plasma glucose, Total Cholesterol (TC), Triglyceride (TG) and High Density Lipoprotein Cholesterol (HDL-C) levels, C peptide, blood pressure measurement and kidney parameter details, which will include test on albuminuria, if done, will be recorded on the CRF. All the anthropometric measurements, if done, will also be recorded. Thyroid function tests, if done, to test for hypothyroidism or hyperthyroidism, if it is per routine clinical practice will be recorded in the CRF.

For analysis, the current smokers and ex-smokers will be categorized in the “ever smokers” group. Similarly, the current tobacco chewer and ex-tobacco chewer will be categorized in the “ever tobacco chewers” group. Ever smoker and ever tobacco chewer groups will be considered as tobacco users. Current alcohol users will be defined as subjects who had consumed alcohol at least once in the last 1 month period. The main occupational level will be divided into three categories: Low (e.g., skilled workers, household workers and retired); medium (e.g., desk jobs) and high (e.g., professionals and businessmen). Physical activity will be categorized as sedentary (sitting, standing and driving for most of the day, cooking, light cleaning, light yard work, slow walking and other major activities involve sitting); moderate (an occupation that includes lifting, lots of walking or other activities that keep you moving for several hours qualifies as moderately active) and heavy (heavy manual labor, a very active lifestyle, dancer or very active sports played for several hours almost daily, an elite athlete in training or an extremely active lifestyle - both at work and at play and sport or activity.

3.2 Design:

Observational, non-interventional, multi-center, real world study in a naturalistic setting. Blood pressure (BP) will be recorded, per routine clinical practice, after the subjects have rested for at least five minutes. Three readings will be taken five minutes apart and the average of three readings will be considered as the BP. Hypertension will be diagnosed based on drug treatment for hypertension or if the BP was >140/90 mmHg according to the Indian Guidelines for Hypertension-4 criteria. The diagnosis of diabetes mellitus will be done using criteria established by the American Diabetes Association. Either fasting plasma glucose (FPG) level ≥126 mg/dl after a minimum 12-hour fast, or 2-hour post glucose level (≥200 mg/dl). National Cholesterol Education Program (NCEP) guidelines will be used for definition of dyslipidemia as presence of ≥1 abnormal serum lipid concentrations such as hypercholesterolemia, high LDL-C, hypertriglyceridemia and low HDL-C. Body mass index (BMI) values defined according to the recommendations of Indian Council of Medical Research (ICMR) for Indians will be used. A study subject will be considered to be obese if BMI is ≥25 kg/m2, and overweight when BMI was 23-24.9 kg/m2. The criteria for glycemic status would be HbA1c <7% (good control), 7-8% (suboptimal control), 8-9% (inadequate control) and >9% (uncontrolled).

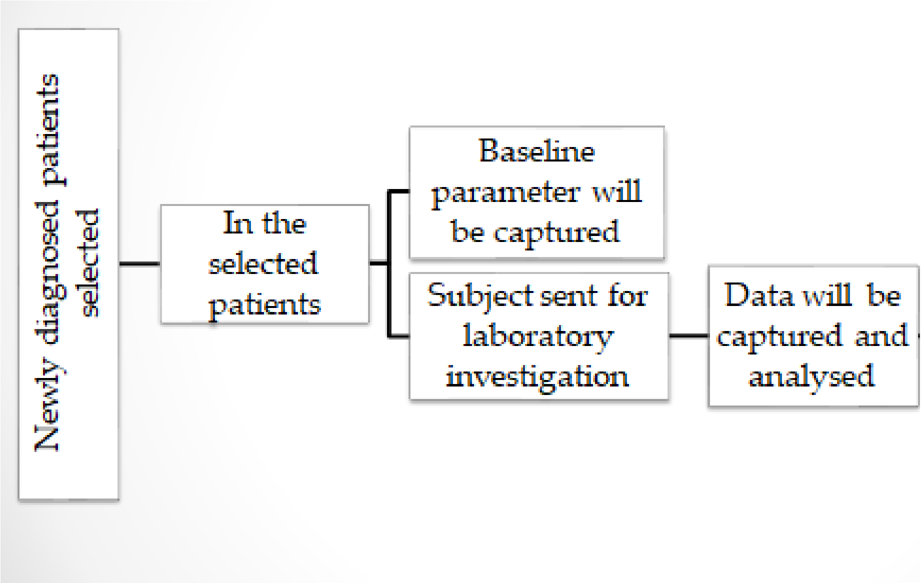

4. CONSENT OF THE INVESTIGATOR:

A written consent of the investigator will be taken before the initiation of the study. If the participant is incapable of giving an informed consent, the participant's legally acceptable representative will sign the consent. If the participant or his legally acceptable representative is unable to read/write, an impartial witness will be required who will be present during the entire informed consent discussion and will also sign the consent form. The formal consent of a participant, using the IEC approved consent form, will be obtained before that participant undergoes screening or any study procedure.

Before requesting such consent, the investigator will provide all essential information for participants to make an informed decision about their participation in a language that is non-technical and understandable by the participant and/or his/her legally acceptable representative.

5. INCLUSION & EXCLUSION CRITERIA:

5.1 Inclusion Criteria:

Subjects meeting the following criteria will be included in the study:

- Adult patients (Aged 18 Years or above) who were newly diagnosed with type 2 diabetes and referred to the clinic within 1 year of diagnosis.
- Diagnosis of diabetes will be based on American Diabetes Association criteria.

5.2 Exclusion criteria:

Subjects meeting the following criteria will be excluded in the study:

- Monogenic diabetes syndrome
- Disease of the exocrine pancreas
- Gestational Diabetes
- Acute or severe chronic heart disease
- Renal or psychiatric disease

6. STUDY PROCEDURES

6.1 SCREENING VISIT (VISIT 0/ ENROLLMENT VISIT)

- All the information should be obtained at one visit
- Informed Consent will be retrieved from the subjects
- Relevant participant data (Clinical and demographical, treatment, etc.) will be collected from the clinic data base

7. STUDY EVALUATIONS AND MEASUREMENTS

7.1 Baseline parameter

Patient's socio-demographic details, family history, concomitant risk factors, medication history, and whatever the investigator captures as part of routine clinical practice

7.2 Laboratory investigation

Anthropometric parameters, fasting plasma glucose, post prandial plasma glucose, HbA1c, lipid profile, urinalysis (for albumin), C-Peptide.

7.3 Screening and Monitoring Evaluations and Measurements

It must be aimed to obtain the following subject data -

- Medical Record Review, demographic, clinical parameters, family and social history

7.4 Laboratory Evaluations

Laboratory evaluation will be done through a standard lab. Specific laboratory results will be documented with the use of the available data from the subjects' file.

8. STATISTICAL CONSIDERATIONS

8.1 Statistical Methods

All subjects included for analysis must provide valid data and thus no methodology for replacing missing data will be implemented. Main demographic and clinical data of subgroups will be summarized by calculating the mean (±SD) in case of continuous variables and the absolute (n) and relative (%) frequency in case of categorical variables.. Results will be presented in P-values and 95% Confidence Intervals (95CI). A p value of <0.05 will be considered significant. Data analysis will be performed using IBM SPSS Statistics version 24 for Windows.

8.2 Clinical Adverse Events

The present proposal entails an observational study which means that AEs are most unlikely caused by the design of the study. However, Adverse Events (AEs) will be captured throughout the study as part of routine spontaneous AE reporting.

Adverse Event Reporting

Since the study procedures are not greater than minimal risk, SAEs are not expected. If any unanticipated problems related to the research involving risks to subjects or others happen during this study, (including SAEs) these will be reported, according to current regulatory requirement, per spontaneous AE reporting process.

Definition of an Adverse Event

An adverse event is any untoward medical occurrence in a subject who has received an intervention (drug, biologic, or other intervention). The occurrence does not necessarily have to have a causal relationship with the treatment. An AE can therefore be any unfavourable or

unintended sign (including an abnormal laboratory finding, for example), symptom, or disease temporally associated with the use of a medicinal product, whether or not considered related to the medicinal product.

All AEs (including serious AEs) will be recorded on the case report form with a full description including the nature, date and time of onset, determination of non-serious versus serious, intensity (mild, moderate, severe), duration, causality, and outcome of the event.

8.3 Definition of a Serious Adverse Event (SAE)

An SAE is any adverse drug experience occurring at any dose that results in any of the following outcomes:

- Death,
- A life-threatening event (at risk of death at the time of the event),
- Requires inpatient hospitalization or prolongation of existing hospitalization,
- A persistent or significant disability/incapacity, or
- A congenital anomaly/birth defect in the offspring of a subject.

Important medical events that may not result in death, be life-threatening, or require hospitalization may be considered a serious adverse drug event when, based upon appropriate medical judgment, they may jeopardize the subject and may require medical or surgical intervention to prevent one of the outcomes listed in this definition.

A distinction should be drawn between serious and severe AEs. A severe AE is a major event of its type. A severe AE does not necessarily need to be considered serious. For example, nausea which persists for several hours may be considered severe nausea, but would not be an SAE. On the other hand, a stroke that results in only a limited degree of disability may be considered a mild stroke, but would be an SAE.

9. STUDY ADMINISTRATIONS

9.1 Data Collection and Management

The study case report form (CRF) is the primary data collection instrument for the study. All data requested on the CRF must be recorded. All missing data must be explained. If a space on the CRF is left blank because the procedure was not done or the question was not asked, write "N/D". If the item is not applicable to the individual case, write "N/A". All entries should be printed legibly. If any entry error has been made, to correct such an error, draw a single straight line through the incorrect entry and enter the correct data above it. All such changes must be initialled and dated. DO NOT ERASE OR WHITE OUT ERRORS. For clarification of illegible or uncertain entries, print the clarification above the item, then initial and date it. Electronic form of case report forms (e-CRFs) will be used as and when required.

9.2 Data sources (if applicable, for existing records)

Source data is all information, original records of clinical findings, observations, or other activities in a study necessary for the reconstruction and evaluation of the study, Source data are contained in source documents. Examples of these original documents, and data records includes (but not limited to): hospital records, clinical and office charts, laboratory notes, memoranda, subject's diaries or evaluation checklists, pharmacy dispensing records, recorded data from automated instruments, copies or transcriptions certified after verification as being accurate and complete, microfiches, photographic negatives, microfilm or magnetic media, x-rays, subject files, and records kept at the pharmacy, at the laboratories, and at medico-technical departments involved in the non-interventional study.

9.3 Confidentiality

Information that can identify study subjects will be kept confidential and managed according to the requirements of the applicable law(s). Subject authorization to collect protected health information (PHI) will be a part of informed consent process. Data privacy clause will be the main aspect of such real world study ICFs.

In the event that a subject revokes authorization to collect or use PHI, the investigator, by regulation, retains the ability to use all information collected prior to the revocation of subject authorization. For subjects that have revoked authorization to collect or use PHI, attempts should be made to obtain permission to collect at least safety information at the end of their scheduled study period.

10 REGULATORY AND ETHICAL CONSIDERATIONS

10.1 Data Safety Monitoring Plan

This study will be monitored sufficiently to ensure that study is conducted, recorded and reported in accordance to approved observation plan, GCP, institutional policies and locally applicable regulatory requirements. The investigator will allocate adequate time for such monitoring activities. The Investigator will also ensure that the monitor or other compliance or quality assurance reviewer is given access to all the above noted study-related documents.

10.2 Risk Assessment

Since the study is designed as observational study, estimated risk for participants is not greater than minimal risk.

10.2.1 Potential Benefits of Study Participation

As this is an observational study, there will be no direct benefits to the study participants. However, data generated will be useful to the physicians in diagnosis and treatment of future subjects.

10.2.2 Risk-Benefit Assessment

Since the study is designed as observational study, estimated risk for participants is not greater than minimal risk. Data generated will be useful to the physicians in diagnosis and treatment of future subjects.

10.3 Recruitment Strategy

Participating physicians will primarily be responsible for recruitment from the patients visiting them.

10.4 Informed Consent

The Investigator shall obtain a freely given, informed and written consent by the subject. If the subject is incapable of giving an informed consent, the subject’s legally acceptable representative will sign the consent. If the subject or his legally acceptable representative is unable to read/ write, an impartial witness will be required who will be present during the entire informed consent discussion and will also sign the consent form. The formal consent of a subject, using the IEC-approved consent form, will be obtained before that subject undergoes any study procedure.

Before requesting such consent, Investigator will provide all essential information for subjects to make an informed decision about their participation (as required by current regulatory norms) in a language that is non-technical and understandable by subject and/or his legally acceptable representative.

10.5 Payment to Subjects/Families

Since the study is designed as an observational, real world, non-interventional study in the naturalistic setting of routine or typical clinical practice, no payment will be made to subjects for participation in this study. Compensation also does not apply to such studies.

11. REFERENCES

- Indian Journal of Clinical Practice, Vol. 24, No. 2, July 2013
- Endocrine 2013; 44:66–9
- Lancet Diabetes Endocrinol 2017 July 22, 2019
- Diabetes Res ClinPract 1995; 28:103–17.
- Indian J Med Res 125, March 2007, pp 217-230
- BMJ Open Diab Res Care 2018;6:e000568
- Indian Journal of Endocrinology and Metabolism, Volume 21; Issue 3; May-June 2017
- Tripathy et al. Diabetol Metab Syndr (2017) 9:8
- Lancet Diabetes Endocrinol 2019 July 22, 2019
- Medical anthropology 2017, VOL. 36, NO. 2, 96–110
- Diabetes care, volume 36, supplement 2, august 2013
- J Med Res 2007; 125(3):275-96
- Ahrén B, et al. Diabetes Obes Metab 2011; 13:775–83
- Diabetologia 2006;49(6):1175-8
- Indian Journal of Clinical Practice, Vol. 22, No. 8, January 2012
- https://www.idf.org/aboutdiabetes/what-is-diabetes/facts-figures.html

Subject ID.: | | | | | | | | | 0 | 1 | Subject Initial: | | | |

Case Report Form (Baseline/enrollment Visit)

Study No. ERIS/OS/20/001   **Version No.:** 01   **Dated:** D D M M Y Y Y Y

India Diabetes Study (IDS)

"A cross-sectional (baseline) & observational real world study on newly diagnosed patients with type 2 diabetes in India."

Investigator Name : ______________________

Site Name and Address : ______________________

Central Laboratory Name : ______________________

Site Code: : | | | | | | | | |

General instructions for CRF filing:

- Entries in the CRF are to be made using Black ink ballpoint pen(preferably).
- Ensure all the entries are to be made accurately, legible, and verifiable with the source data.
- In case of corrections:
  - ◊ Do not over right or erase
  - ◊ Do not use corrections materials (Whiteners, cello tapes, bleaching).
  - ◊ Delete the incorrect entry with single line over it without obscuring original entry text, write the correct information nearby with dated sign If required, explain reason for correction.
  - ◊ Dates should be recorded (preferably) as DD/MM/YYYY.
  - ◊ Enter 3 letters Subject initials considering first letter of First Name/ Middle Name /Last Name (e.g. SKN for Suresh Krishna Nagraj). In case of absence of middle name, initial should be written as first 02 letters of First name and first letter of Last Name (e.g. SU for Suresh Nagraj).
- Acceptable abbreviations are the following:
  - ◊ Unknown: UNK, Not Done: ND & Not Applicable: NA, Not Available-Not available
- Wherever required, always use "√" a tick mark symbol for choosing the appropriate answer.
- For Inclusion and Exclusion Criteria, put "√" in YES/NO/NA column, as applicable.
- Avoid the use of symbols or abbreviations for medical terminologies.
- Please do not leave any fields blank. The answer to any question if not known or unavailable mention "Unknown" or UNK or not available and if not applicable mention NA.
- Any errors should be stricken out with a single line so that original entry is not obscured; the new entry made above or adjacent should be dated signed.
- Enter Site Number according to the number/instruction provided by Sponsor/Sponsor's representative.
- Assign 10 digits Subject ID number in chronological order serially. Among ten-digit number, first eight digits stand for site number and last two digits for serial no. of subject at a particular participant hospital. e.g. for Site Number 00000001, Subject ID will be e.g. 0000000101, 0000000102, 0000000103, etc.

Subject ID.: | | | | | | | | 0 | 1 | Subject Initial: | | | |

Visit Type: **Visit 0/Baseline/Enrollment visit**   **Visit Date:** D D M M Y Y Y Y

Written Informed Consent taken: ☐ **YES** ☐ **NO**

Demographics / Anthropometric assessment Instructions:

- If exact birth date is not known, then at least birth year MUST be entered. (E.g. 01/Jan/1965). Every effort should be made to capture the date as accurate as possible.
- Always mention age in completed years.

**Birth Date (DD/MM/YYYY)**	D	D	M	M	Y	Y	Y	Y
**Age (in completed years)**	☐☐							
**Gender**	Male ☐ Female ☐							
**Height (in m)**								
**Weight (in kg)**								
**BMI (kg/m^2)**								

**Physical Activity**	Sedentary ☐ Moderate ☐ Heavy ☐

1. Sedentary (sitting, standing and driving for most of the day, cooking, light cleaning, light yard work, slow walking and other major activities involve sitting).
2. Moderate (an occupation that includes lifting, lots of walking or other activities that keep you moving for several hours qualifies as moderately active)
3. Heavy (heavy manual labor, a very active lifestyle, dancer or very active sports played for several hours almost daily, an elite athlete in training or an extremely active lifestyle - both at work and at play and sport or activity

Social History:

**Smoking**	Yes ☐ No ☐ Ex-Smoker ☐
**Other Tobacco products Consumption**	Yes ☐ No ☐ Ex-tobacco chewer ☐
**Does subject has history of alcohol in last one month?**	Yes ☐ No ☐
	**If Yes,** Regular ☐ Occassional ☐

Food Habit: Veg ☐ Non-Veg ☐

Subject ID.: | | | | | | | | 0 | 1 | **Subject Initial:** | | | |

Diabetes Family History:

**Mother**	Yes ☐ No ☐ Unknown ☐
**Father**	Yes ☐ No ☐ Unknown ☐
**Number of Siblings**	☐
**How many are type 2 Diabetic patients**	☐

Sleep Duration: Less than 6 ☐ 6 - 9 Hours ☐ More than 9 Hours ☐

Medical History and Concurrent Illness (Associated Co-morbidities):

Sr. No.	Associated co-morbidity	Present (Yes/No)	If yes, Start Date (DD-MM-YYYY)	Comments (If any)
1	Hypertension			
2	Dyslipidemia			
3	Heart disease			
4	Other:____________			

Note: **1.** If the subject is consuming any medication for the condition mentioned above, then enters the details in Concomitant Medication Form. **2.** ecord medications ONLY relevant to Diabetes and Co-morbidities illness(es) management. **3.** If exact date is not known, then at least year MUST be entered

Vital Signs:

Parameters	Readings
**Pulse Rate (beats per min)**	☐☐☐
**Blood Pressure (mmHg)**	SBP ☐☐☐ DBP ☐☐☐

Medication History and Concomitant Medication(s):

- Instructions for completion of medication history and concomitant medications
- Record medications relevant to Diabetes and Co-morbidities illness(es).
- Provide generic names for medications where possible. When the generic name is not known or the medication is a combination product, provide the trade name.
- In case of fixed dose combinations, please capture all its generic components.
- Please record only one medicine per line.
- Please do not use abbreviations for the generic name of medication

Subject ID.:									0	1

Subject Initial:			

Sr. No.	Medication		Strength	Dose (OD / BID / TID)	Start date (dd/mm/yyyy)	End date (dd/mm/yyyy)
	Generic Name	Route (e.g., Oral, I.V., I.M., S.C.)				

Inclusion Criteria:

Sr. No.	Inclusion Criteria (Please mark √ in the relevant boxes against each inclusion criteria)	YES	NO
1	Adult patients (Aged 18 Years or above) who were newly diagnosed with type 2 diabetes and referred to the clinic within 1 year of diagnosis	☐	☐
2	Diagnosis of diabetes will be based on American Diabetes Association criteria.	☐	☐

Exclusion Criteria:

Sr. No.	Exclusion criteria (Please mark √ in the relevant boxes for each exclusion criteria)	YES	NO	NA
	**Monogenic diabetes syndrome**	☐	☐	☐
	**Disease of the exocrine pancreas**	☐	☐	☐
	**Gestational Diabetes**	☐	☐	☐
	**Acute or severe chronic heart disease**	☐	☐	☐
	**Renal or psychiatric disease.**	☐	☐	☐
	**Initial evaluation in clinic after 1 year of diagnosis or unclear at time of diagnosis, type 1, type 1.5, or type 3 diabetes**	☐	☐	☐

Note: If any of the Exclusion criteria marked as "YES" and/or Inclusion criteria as "NO", then, the patient will not be eligible for the study.

Subject ID.: | | | | | | | | | 0 | 1 | **Subject Initial:** | | | |

Laboratory Investigations:

Date of sample collection: D D M M Y Y Y Y

Parameter	Value	Unit	Normal	Abnormal
**BLOOD GLUCOSE:**				
Fasting Plasma Glucose		mg / dL	☐	☐
Postprandial Plasma Glucose		mg / dL	☐	☐
HbA1c		%	☐	☐
C-Peptide		ng / mL	☐	☐
**LIPID PROFILE:**				
Total Cholesterol (TC)		mg / dL	☐	☐
LDL Cholesterol		mg / dL	☐	☐
HDL Cholesterol		mg / dL	☐	☐
Triglycerides (TG)		mg / dL	☐	☐
**KIDNEY PROFILE:**				
Serum Creatinine		mg / dL	☐	☐
**THYROID PROFILE:**				
T3		ng / dL	☐	☐
T4		μg / dL	☐	☐
TSH		μIU / mL	☐	☐

Recorded by:

Name ____________________ Date & Sign ____________________ Role ____________________

Reviewed and approved by (Study Physician):

Name ____________________ Date & Sign ____________________ Role ____________________
